# Supplementary material for: Quantum critical phase of FeO spans conditions of Earth’s lower mantle
Source: Nat Commun. 2024 Apr 24;15:3461. doi: 10.1038/s41467-024-47489-w (PMC11043421; doi:10.1038/s41467-024-47489-w)
Supplement: Supplementary file 1 — Supplementary Information [file 41467_2024_47489_MOESM1_ESM.pdf]

Supplementary Materials for: Quantum critical phase of FeO spans conditions of Earth's lower mantle

Wai-Ga D. Ho,[1] Peng Zhang,[2] Kristjan Haule,[3] Jennifer M. Jackson,[4] Vladimir Dobrosavljević,[1] and Vasilije V. Dobrosavljevic[4,5]

[1]*Department of Physics and National High Magnetic Field Laboratory, Florida State University, Tallahassee, FL, USA*
[2]*MOE Key Laboratory for Non-equilibrium Synthesis and Modulation of Condensed Matter, Shaanxi Province Key Laboratory of Advanced Functional Materials and Mesoscopic Physics, School of Physics, Xi'an Jiaotong University, 710049, Xi'an, Shaanxi, P.R.China*
[3]*Center for Materials Theory, Department of Physics, Rutgers University, Piscataway, NJ, USA*
[4]*Seismological Laboratory, California Institute of Technology, Pasadena, CA, USA*
[5]*Earth and Planets Laboratory, Carnegie Institution for Science, Washington, D.C. 20015, USA*

I. THE EDMFT ALGORITHM

In density functional theory (DFT) the functional of charge density $\rho(\mathbf{r})$ is optimized, in which the effective single-particle potential is derived from the properties of the uniform electron gas. Similarly, in DFT+Embedded DMFT method the Klein functional [1] is extremized. This functional depends on the single-particle Green's function $G(\mathbf{r},\mathbf{r}',\omega)$ and can be expressed as follows:

$$\Gamma[\{G\}] = \mathrm{Tr}(\log G) - \mathrm{Tr}((G_0^{-1} - G^{-1})G) + E^{H+XC}_{V_c}[\{\rho\}] + \Phi^{DMFT}_{V_{DMFT}}[\{G_{loc}\}] - \Phi^{DC}_{V_{DMFT}}[\{\rho_{loc}\}]. \tag{S1}$$

Here $G(\mathbf{r},\mathbf{r},\tau=0^-) = \rho(\mathbf{r})$ represents the charge density, $G_0^{-1}(\omega) = \omega + \mu + \frac{\nabla^2}{2m} - V_{ext}$ is the non-interacting Green's function, containing only the external potential of the crystal. The term $E^{H+XC}_{V_c}$ corresponds to the DFT Hartree and exchange-correlation energy functional. The Hartree term is treated exactly, while the exchange-correlation term is derived from the properties of the uniform electron gas. If the last two terms in the functional are removed, extremizing the Klein functional leads precisely to the DFT equations. The value of the functional at the extreme is the free energy of the system, equal to the DFT total energy at $T=0$. It's worth noting that in the Klein functional interpretation, the Kohn-Sham bands are not auxiliary fictitious states without physical significance. Instead, they are approximate spectra obtained by approximating the exact functional $\Phi[G]$ with $\Phi[G] \approx E^{H+XC}_{V_c}[\rho]$. In exact Klein theory, the $\Phi[\{G\}]$ is the sum of all skeleton Feynman diagrams, obtained by exact G and the Coulomb interaction V_c.

In the embedded DMFT approach, local Feynman diagrams denoted by $\Phi^{DMFT}_{V_{DMFT}}[\{G_{loc}\}])$ are added to the DFT exchange-correlation functional. Its important to emphasize that this addition is expected to enhance the precision of the Φ functional when compared to LDA/GGA. This improvement arises from the transition from the constraint of locality to a point in 3D space within LDA/GGA, to locality to an atom within the solid in DMFT. The local DMFT diagrams needed in Φ^{DMFT} are constructed using the single-electron propagator G_{loc} and the screened Coulomb interaction $V_{DMFT}(\mathbf{r})$. The local propagator is defined as the projection of the single-particle Green's function onto quasi-atomic orbitals:

$$G_{loc}(\mathbf{r},\mathbf{r}') = \sum_{\alpha,\beta} \langle \mathbf{r}|\phi_\alpha\rangle \langle\phi_\alpha|G|\phi_\beta\rangle \langle\phi_\beta|\mathbf{r}'\rangle. \tag{S2}$$

In this equation, $\phi_\alpha(\mathbf{r})$ represents the solutions of the Schroedinger equation within the muffin-tin sphere, linearized at the Fermi level. In the current calculation, the sum over $\alpha\beta$ extends over iron $3d$ orbitals.

The matrix elements of the Coulomb interaction $V_{DMFT}(\mathbf{r})$ within the $3d$ localized orbitals have the Slater form:

$$U_{m_1,m_2,m_3,m_4} = \sum_{m,k} \frac{4\pi}{2k+1} \langle Y_{lm_1}|Y_{km}|Y_{lm_4}\rangle \langle Y_{lm_2}|Y^*_{km}|Y_{lm_3}\rangle F^k. \tag{S3}$$

Here, $l=2$ corresponds to the $3d$ orbitals, and m and k range from -2 to 2. The Slater integrals are associated with the screened interaction and are parametrized as $F^0 = U$, $F^2 = 112/13J$, and $F^4 = 70/13J$. The parameter U represents the onsite Hubbard interaction, while J is known as the Hund's interaction. These values are obtained via constrained-eDMFT calculations, wherein a supercell is constructed, and the charge density is varied on a single-site. The charging energy, related to these screened parameters, is deduced from the total energy of the eDMFT functional.

In the current work, we use an Ising-type interaction, which speeds up the algorithm substantially. The Ising interaction is constructed as follows: i) we first transform the Coulomb matrix elements from spherical harmonics basis U_{m_1,m_2,m_3,m_4} to the real harmonics basis $(xz, yz, xy, z^2, x^2-y^2)$, which we denote by $U_{i,j,k,l}$. In this basis the Green's function is diagonal. ii) We than

approximate $U_{i,j,k,l}$ with terms that involve at most two different orbitals, i.e., $U_{i,j,j,i}$, $U_{i,j,i,j}$, $U_{i,i,j,j}$. When two or four different orbitals are involved, the term is discarded.

The double-counting functional $\Phi^{DC}_{V_{DMFT}}[\{\rho_{loc}\}]$ has been derived in Ref. [2]. It closely resembles the DFT functional, but the charge density in this functional is projected onto the $3d$ orbitals only ($\rho_{loc}(\mathbf{r}) = G_{loc}(\mathbf{r},\mathbf{r},\tau = 0^-)$), and the bare Coulomb repulsion $V_c(\mathbf{r}) = \frac{1}{4\pi\varepsilon_0 \mathbf{r}}$ is replaced by the screened Coulomb repulsion $V_{DMFT}(\mathbf{r})$. The functional is given by:

$$\Phi^{DC}_{V_{DMFT}}[\{\rho_{loc}\}] = \frac{1}{2}\int d\mathbf{r}d\mathbf{r}'\rho_{loc}(\mathbf{r})V_{DMFT}(\mathbf{r}-\mathbf{r}')\rho_{loc}(\mathbf{r}') + \int d\mathbf{r}\rho_{loc}(\mathbf{r})\varepsilon^{V_{DMFT}}_{xc}[\{\rho_{loc}\}] \tag{S4}$$

where $\varepsilon^{V_{DMFT}}_{xc}[\{\rho_{loc}\}]$ is obtained from the solution of the uniform electron gas problem, as in DFT. However, in this functional, the interaction is screened rather than the bare Coulomb interaction. For more details, we refer the reader to Ref. [3].

II. FLUCTUATING MAGNETIC MOMENT, OCCUPATION NUMBER, AND OPTICAL CONDUCTIVITY

The fluctuating local magnetic moment is closely linked to the Curie-Weiss moment and is defined as: $M = g\sqrt{\langle S_z^2\rangle}$. This quantity can be evaluated using the impurity solver, which computes the probabilities of atomic states, denoted as P_k. Specifically, it can be expressed as: $M = 2\sqrt{\sum_{\mathbf{k}} P_k S_k^{z\,2}}$. Here k serves as an index referring to the many-body states of the iron $3d$ shell.

The occupation number of the correlated Fe-3d-orbitals is defined by $n = \sum_{\alpha\sigma}(G_{loc}(\tau = 0^-))_{\alpha,\sigma}$, where α and σ are the orbital and spin indices.

The optical conductivity is calculated as follows [4]:

$$\sigma^{\mu,\nu}(\omega) = \frac{\pi}{V}\sum_{\mathbf{k}}\int_{-\infty}^{\infty} d\varepsilon \frac{f\left(\varepsilon - \frac{\omega}{2}\right) - f\left(\varepsilon + \frac{\omega}{2}\right)}{\omega} \mathrm{Tr}\left[A_{\mathbf{k}}\left(\varepsilon + \frac{\omega}{2}\right)\mathbf{v}^{\mu}_{\mathbf{k}} A_{\mathbf{k}}\left(\varepsilon - \frac{\omega}{2}\right)\mathbf{v}^{\nu}_{\mathbf{k}}\right] \tag{S5}$$

where V is the volume, $\mathbf{v}^{\mu}_{\mathbf{k}}$ are the velocities computed by $(v^{\mu}_{\mathbf{k}})_{n,m} = \frac{e\hbar}{m}\langle\psi_{n\mathbf{k}}| - i\partial_\mu|\psi_{m,\mathbf{k}}\rangle$, μ, ν represent the Cartesian coordinates (x,y,z), $A_{\mathbf{k}}(\varepsilon) = \frac{1}{2\pi i}(G^{\dagger}_{\mathbf{k}}(\varepsilon) - G_{\mathbf{k}}(\varepsilon))$ is the matrix of spectral functions written in the basis of Kohn-Sham orbitals $\psi_{n\mathbf{k}}$, $f(\varepsilon) = \left(e^{\beta\varepsilon}+1\right)^{-1}$ is the Fermi-Dirac distribution function, and the trace is taken over all valence Kohn-Sham states. The DC electrical conductivity is extracted in the $\omega \to 0$ limit.

III. MOTT QUANTUM CRITICALITY

Critical points are generally associated with second-order phase transitions [6], where the characteristic length-scales and time-scales diverge, and all thermodynamic and transport properties display scaling behaviors. This paradigm was well established for conventional (thermal) phase transitions, where classical models suffice [6]. More recent work focused on *quantum phase transitions* [5], which are found at $T = 0$ where quantum effects cannot be ignored. Due to the Heisenberg Uncertainty Principle linking time scales and energy scales ($t \sim \hbar/E$), here one expects [5] the emergence of a "crossover" temperature T^* that characterizes each phase, which vanishes as the quantum critical point (QCP) is approached. The typical phase diagram then assumes a generic form shown in Fig. S1. This behavior has been well established [5] around many QC points, especially in familiar cases where the QC point marks the boundary of a given symmetry-broken phase (e.g. ferromagnetic or antifferomagnetic order, superconductivity, charge density wave order, etc.).

Behavior around metal-insulator transitions (MIT) is more complicated, because in many cases these two phases do not differ by any static symmetry, and there exists a sharp MIT only at $T = 0$ [7]. Nevertheless, in many cases the experiments provide evidence for distinct quantum criticality, including the expected scaling behavior of the resistivity curves [8]. The specific case of the Mott MIT is even more controversial, because bandwidth (pressure) driven Mott transitions often display "weakly first-order" character, with a first-order transition and the associated phase coexistence region at low temperatures below a critical end-point $T = T_c$. On the other hand, microscopic calculations [9–11], as well experiments [12, 13] have established that $T_c \ll T_F \sim U$, while a well-defined QC behavior arises in a broad region at $T_c < T \ll T_F$. Characteristic metallic behavior (e.g. T^2 resistivity expected from Fermi Liquid theory) arises on the metallic side, but only below a characteristic "Brinkmann-Rice" temperature marking the thermal destruction of Landau quasiparticles $T_{BR} \sim T_{FL}$ [14, 15], while the familiar activated transport emerges [16] on the insulating side at $T < T_{gap}$, with the activation energy T_{gap} being of the order of the Mott gap. The two temperature scale T_{BR} and T_{gap}, which are both seen to decrease towards a transition [13, 14], mark the boundary of an intermediate QC region, where all physical observables are expected to display a characteristic scaling behavior.

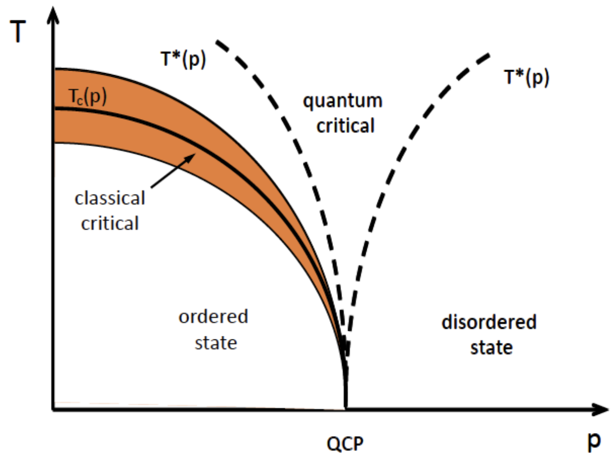

FIG. S1. Generic phase diagram expected for a quantum critical point. For symmetry-broken phases, one generally expects a finite ordering temperature $T_c(p)$, which vanishes at QCP, and classical behavior in its vicinity (shaded region). In addition , one expects one crossover temperature line on each side of the transition, marking the boundary of the quantum critical (QC) region, which broadens as temperature increases. The behavior within the QC region is dominated by quantum flucuations, and is qualitatively different than the behavior of the respective phases at $T < T^*(p)$.

The notion that such QC transport behavior should emerge above a modest critical end-point temperature of the Mott coexistence region has been first put forward in Ref [10, 11], based on microscopic DMFT theory for a single-band Hubbard model at half-filling. The predicted behavior was soon confirmed by experiments on so-called "spin-liquid" Mott-organics by Kanoda and collaborators [12]. Very similar results were also observed in several other systems displaying Mott (or Mott-like) MITs, including various examples of to-dimensional electron systems in semiconductors (2DEG), as well as certain classes of recently discovered moiré materials [15]; a critical comparison of all these systems, as well as the relevant theoretical pictures was recently given in Ref. [17]. Recent theoretical work [18, 19] further confirmed the existence of Mott QCP, and the predicted scaling behavior of various quantities. What is most remarkable is that almost all *qualitative* aspects of the experimental phenomenology appear to be almost identical in all these systems, although the characteristic energy scales span several orders of magnitude. Specifically, the Fermi temperature in the critical regime T_F is of the order of 10K in 2DEG systems, around 100K in moireé systems, and on the scale of 1000K in Mott organics.

The realization of Mott QC we report for FeO arises on yet one order of magnitude higher energy scale, since here $T_F \sim 3$ x10^4K. On the other hand, as we emphasized, here the QC behavior emerges only in an intermediate *orbitally selective* regime, where only he t_{2g} electrons are becoming mobile, while the e_g electrons still remain Mott-localized. Such orbitally-selective Mott localization is a factor that stabilizes the unfamiliar QC behavior over such a broad range of temperatures and pressures, that it persists over the entire regime relevant to the Earth's core-mantle boundary. The temperatures of relevance, in the range between 2000K and 4000K certainly represent "extreme conditions" on the scale of ambient temperatures, yet this is only about 10% of T_F - precisely the regime where Mott QC was observed in other systems mentioned above!

IV. THERMAL DESTRUCTION OF QUASIPARTICLES AND THE BRINKMAN-RICE LINE

According to Fermi liquid theory [20], even strongly correlated metals should qualitatively behave similarly to an ideal gas of fermions, but only concerning sufficiently low-energy excitations. Here thermodynamic response, as well as transport behavior, is dominated by low-energy *quasiparticle (QP) excitations*. In the presence of strong electronic correlations, these QPs still carry charge e and spin 1/2, but often feature a significantly enhanced effective mass m^*. The corresponding electronic states assume a form of a narrow *QP band*, which produces a sharp *QP peak* [9] in the density of states (DOS), around the Fermi energy. The small spectral weight $Z \sim 1/m^*$ of these QP peaks encodes the extreme fragility of such correlated matter to thermal excitations.

Unlike conventional metals, with characteristic energy on the scale of the Fermi temperature $T_F \sim 10^4$ K, the QP states found in strongly correlated materials can be dramatically affected by much lower temperatures, modifying all observable properties. The characteristic energy scale of these QP states was first estimated theoretically by Brinkman and Rice [21], based on the

Gutzwiller variational approch. The corresponding temperature T_{BR}, at which the QP states are thermally destroyed, is often called the "Brinkman-Rice temperature" [22]. It marks the crossover from the coherent regime dominated by long-lived QP excitations to an incoherent regime, dominated by very strong electron-electron scattering, which we identify with a "quantum critical" (QC) regime [10, 12, 23] associated with the Mott metal-insulator transition.

Most existing theoretical approaches [21] to correlated electronic matter focus on describing the ground state and only the leading low-temperature excitations. They therefore cannot properly describe this coherence-incoherence crossover around $T \sim T_{BR}$, and the physical properties in the regime where thermal excitations dramatically affect the electronic spectra. In contrast, the improved theoretical methods based on DMFT and its extensions [9, 24] are hand-tailored precisely to self-consistently determine the key player in this regime – the electron-electron scattering rate. Physically, when this scattering rate becomes comparable to the characteristic energy scale of quasiparticles, the corresponding electronic states are thermally suppressed. The sharp quasiparticle peak in the DOS spectra then "melts away" [22, 25] in a fairly sudden fashion, which happens at $T \sim T_{BR}$. This behavior can be clearly seen in Fig. S2, where we show the evolution of DOS spectra with increasing temperature, focusing on the "orbitally selective" metal [26] which forms at low temperature as soon as the t_{2g} gap closes. While a very sharp QP peak is seen at $T = 300$ K, it is quickly diminished already at $T = 1000$ K, and it completely disappears at $T = 2000$ K, where it is replaced by a shallow pseudogap feature around the Fermi energy (for comparison with similar behavior in a one-band Hubbard model, see Fig. 7 in Ref. [11]). The corresponding behavior of the self-energies is shown in Fig. S4.

The sudden modification of spectral features with temperature allows us to identify the corresponding crossover temperature T_{BR}, which we define as the temperature where the DOS value at the Fermi energy (the "height" of the QP peak) is suppressed. This procedure has been repeated at each of the values of compression studied, defining the "BR line" we displayed on Fig. 1 (main text). We should emphasize that this BR line is *not* a sharp phase transition but is only a (relatively smooth) crossover between two physically distinct regimes. In our case, it marks the boundary of the incoherent QC regime, which differs significantly from the low-temperature quasiparticle metal. Note that T_{BR} increases with compression (see Fig. 1 of main text), so that the destruction of QP states can also be observed by reducing compression at fixed temperature in such a way to cross the BR line (see Fig. 2 of main text, bottom panels). This strategy is especially useful at higher temperatures (T > 2000 K), where the BR line is seen to suddenly "turn up", due to the enhanced metallization caused by the closing of the e_g gap around $\Delta v = 0.34$. It is interesting to observe how the thermal evolution of both the electronic spectra (see color-coded DOS in Fig. 1 of main text) and the electrical conductivity (see color-coded conductivity in Fig. 4 of main text) closely tracks the BR line in the entire phase diagram. The dramatic "upturn" of the BR line at $T > 2000$ K makes it almost "vertical" on the phase diagram. The net result of all this is a notably weak temperature dependence of all quantities within the QC phase. Remarkably, the geotherm trajectory also is almost "vertical" (i.e. pressure-independent) in the lower mantle region just outside the core-mantle boundary. As a result, both the DOS and the electrical conductivity display almost no T-dependence when plotted along the geotherm line (at 2500 K < T < 3500 K), as shown in Fig. S3.

We should also mention the often-discussed "Fermi liquid regime" (FL), associated with the *leading* low-temperature behavior of quasiparticles, where the conductivity $\sigma(T) \sim T^{-2}$. It is worth stressing that this regime corresponds to $T < T_{FL}$, with T_{FL} that is usually significantly smaller than T_{BR}. The intermediate regime $T_{FL} < T < T_{BR}$ is sometimes described as featuring *"resilient QPs"* [22], where the QP parameters (e.g. the QP weight Z) assume a certain T-dependence, and other properties display deviations from standard FL behavior. Both temperature scales T_{FL} and T_{BR} have been experimentally [27] and theoretically [11, 22] identified in certain Mott systems, and are both seen to decrease towards the Mott transition. In this study, however, we shall not explore the details of such low-T QP behavior, primarily because the corresponding correlated metallic $B1$-FeO phase

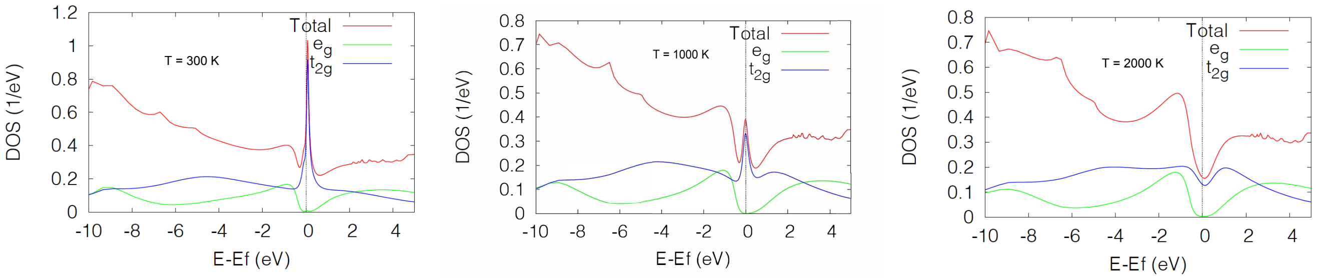

FIG. S2. Electronic DOS of FeO on barely the metallic side of the Mott point ($\Delta v = 0.25$). The left panel shows the spectrum at T = 300K, featuring a sharp QP peak at the Fermi energy, with modest spectral weight. Increasing temperature quickly destabilizes/broadens the fragile QP states, as shown for T = 1000 K (middle panel). The QP states are completely suppressed above the Brinkman-Rice temperature (which is $T_{BR} \approx 1100K$ for this compression), marking the crossover to the QC region at higher temperatures. Here the t_{2g} electronic states are incoherent but already gapless, while the e_g gap remains open, as shown for T = 2000K (right panel).

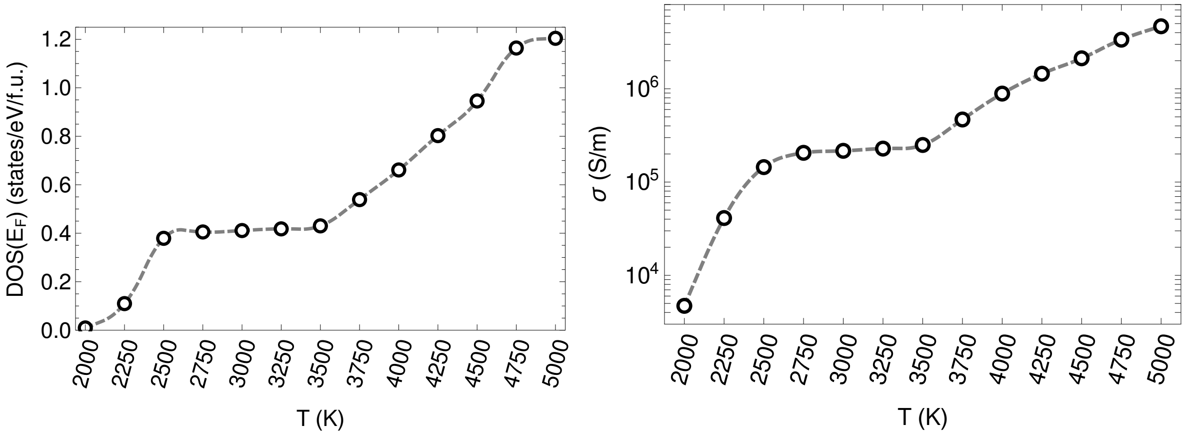

FIG. S3. Both DOS (left panel) and the electrical conductivity (right panel) plotted along the geotherm trajectory. Both display remarkably weak T-dependence within the lower mantle, just outside the core-mantle boundary (2500 K $< T <$ 3500 K).

is not of much direct relevance to the issues surrounding the core-mantle boundary.

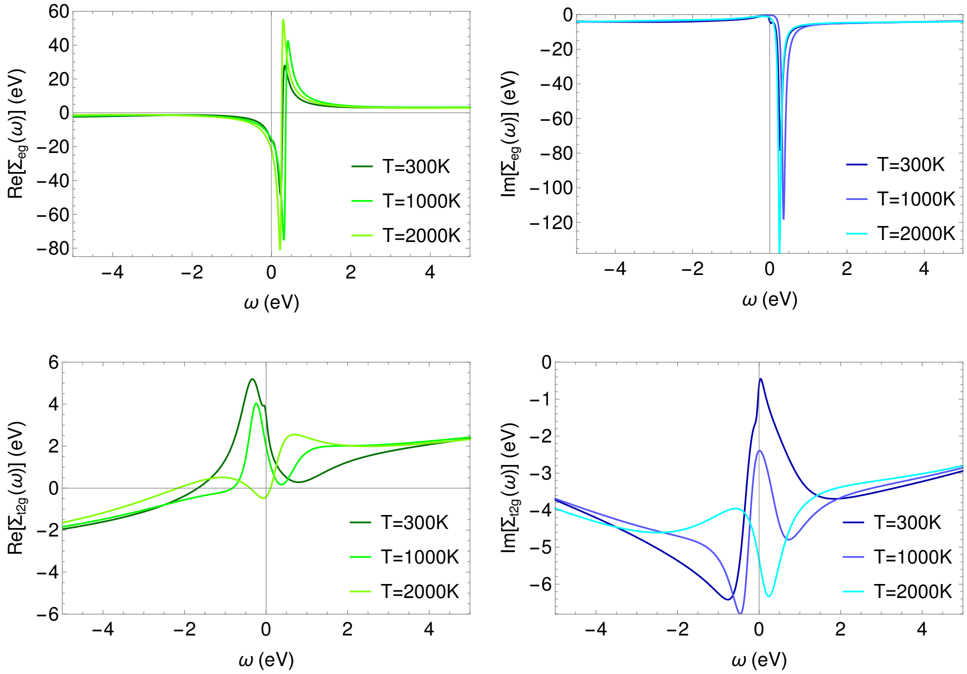

FIG. S4. Self-energies reflecting the DOS data for $\Delta v = 0.25$ and T=300, 1000, 2000 K in Fig. S2. The left/right column contains the real/imaginary part of the self-energy for both the e_g and t_{2g} orbitals (top and bottom rows respectively). In the top two panels, the pole generating the e_g Mott gap is shown to persist for all 300K $\leq T \leq$ 2000K. For the t_{2g} channel, the bottom right panel shows the zero-frequency scattering rate (i.e. magnitude of the self-energy's imaginary part at $\omega = 0$) growing with T as the quasiparticle succumbs to the heat and decoheres (thermal broadening and death of spectral peak near the Fermi energy).

V. ESTIMATING THE MOTT GAP

At ambient conditions FeO is a robust Mott insulator, with a substantial gap to electronic excitations on the scale of several eV. In systems with cubic symmetry (corresponding to the $B1$ *rocksalt* structure), the d-electrons of Fe are distributed between t_{2g} and e_g orbitals which, within a solid, contribute to forming the "Hubbard" bands with corresponding symmetry. Because

crystal field splitting lifts the degeneracy between these orbitals/bands, the corresponding Mott gaps are also in-equivalent. In FeO the t_{2g} band gap is generally smaller than the e_g gap, and it closes first under compression around Δv ≈ 0.22; the e_g gap closes around Δv ≈ 0.43.

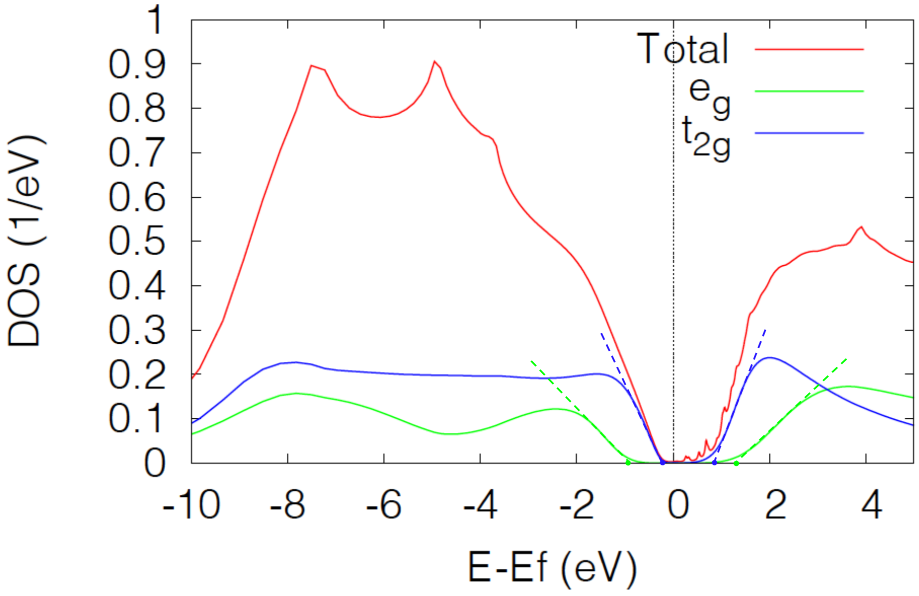

FIG. S5. Density of states (DOS) for FeO under ambient conditions ($P = 0$ GPa, $T = 300$ K) featuring substantial gaps both in the t_{2g} and the e_g orbital/band. The estimate for the low-temperature gap size is performed by linearly extrapolating DOS to zero, towards the band edge.

In order to understand the approach to the IMT, as well as its finite-temperature manifestations, it is important to precisely determine the evolution of these band gaps with compression. The precise definition of the band gap is, however, a bit complicated in our finite-temperature calculations, since thermal excitations generally tend to create band tails and "gap rounding". Still, when the gap size E_g is large as compared to the thermal energy $k_B T$, this effect is modest and a relatively accurate estimation of the $T = 0$ value of the gap is possible. To obtain a quantitative estimate despite such thermal "rounding", we adopt the procedure to linearly extrapolate DOS of a given band, a procedure that can roughly eliminate the rounding effect. This procedure is justified by the fact that, within our DMFT-type setup, the DOS has sharp band edges at $T = 0$; it has also been cross-checked for simple model systems [11], where in certain limits the gap size is accurately know.

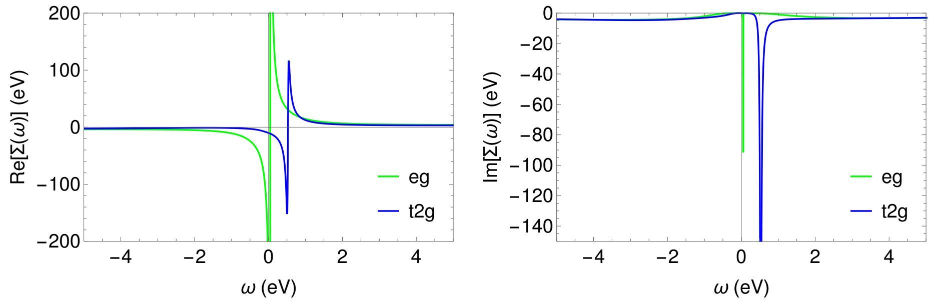

FIG. S6. Self energies reflecting the DOS data for $\Delta v = 0$ and $T = 300$K in Fig. S5 are shown for both orbitals.

To illustrate this procedure, in Fig. S5 we show how the extrapolation is performed under ambient conditions. The corresponding behavior of the self-energies is shown in Fig. S6. This procedure has been repeated for all the values of compression considered, using the results obtained at $T = 300$K, and we obtain the low-temperature value of the given gap energy E_g, as a function of compression. On physical ground and based on previous extensive work on model systems [10, 11], we expect that typical insulating behavior should be observed only at temperatures substantially smaller than the thermal energy corresponding to the gap size. Thus, we define $T_{gap} = E_g/k_B$ as an appropriate temperature scale that marks the boundary

of the insulating region, separating it from the intermediate *gapless* QC region, which displays incoherent transport at finite temperature. Since E_g decreases linearly with compression (for both band gaps), so does the corresponding crossover scale T_{gap}, which vanishes where the $T = 0$ gap does.

This expectation is directly confirmed by plotting T_{gap} as a function of compression on Fig. 1 (main text). Here the DOS at the Fermi energy obtained by our expansive direct computations across the phase diagram is color coded; we observe how DOS assumes (exponentially) small values in the entire region where $T < T_{gap}(\Delta \mathrm{v})$, for given compression. Similar results are obtained in Fig. 4 (main text), where the electrical conductivity is color-coded across the phase diagram, as a function of pressure and temperature. Again, we observe how the conductivity is (exponentially) small in the Mott insulating phase, corresponding to $T < T_{gap}(P)$.

VI. DATA GRID

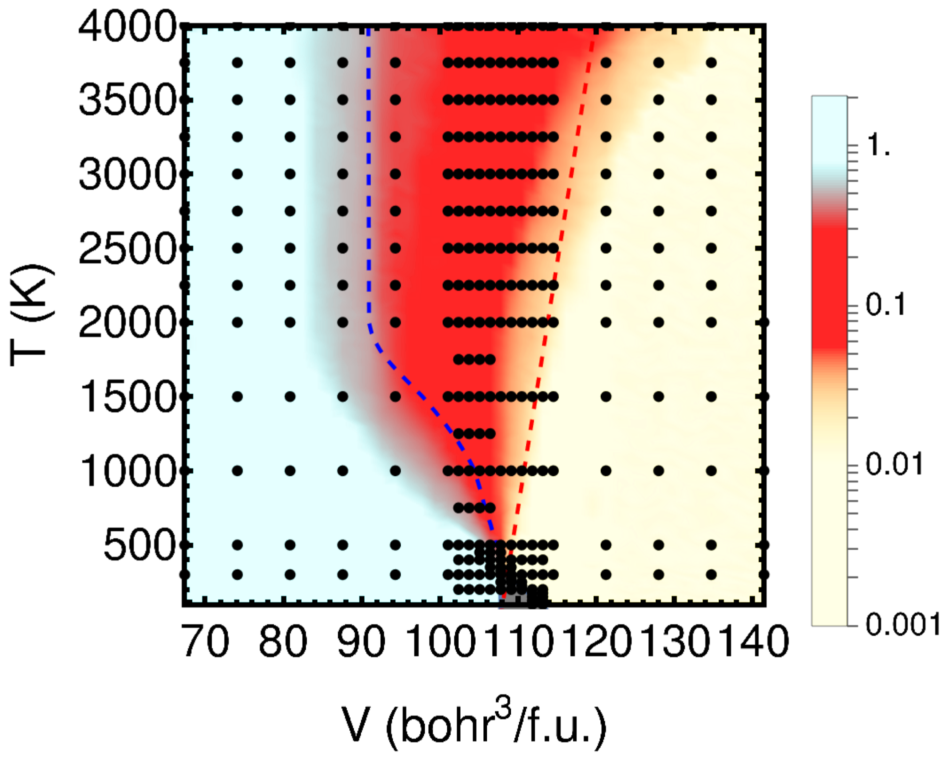

FIG. S7. Phase diagram of $B1$-FeO, with color-coded values of DOS at the Fermi energy. Black dots indicate the grid of points where we solved the eDMFT equations.

While most studies in the past obtained accurate DFT+DMFT data only for a few values of temperatures and volume (pressure), we have performed an expansive set of calculations, canvassing the entire phase diagram of $B1$-FeO. We solved the eDMFT equations on a dense grid of points across the insulator to metal transition (IMT) region. In doing so, we selected a finer grid around the critical volume, since this is where all physical quantities display a somewhat stronger dependence on volume/pressure, as shown on Fig. S7. The color coded values of DOS and the electrical conductivity were obtained by appropriate spline procedures to interpolate between the results explicitly obtained at these data points.

SUPPLEMENTARY REFERENCES

[1] Klein, A. Perturbation theory for an infinite medium of fermions. ii. *Phys. Rev.* **121**, 950–956 (1961).

[2] Haule, K. Exact double counting in combining the dynamical mean field theory and the density functional theory. *Phys. Rev. Lett.* **115**, 196403 (2015).
[3] Haule, K. Structural predictions for correlated electron materials using the functional dynamical mean field theory approach. *Journal of the Physical Society of Japan* **87**, 041005 (2018).
[4] Basov, D. N., Averitt, R. D., van der Marel, D., Dressel, M. & Haule, K. Electrodynamics of correlated electron materials. *Rev. Mod. Phys.* **83**, 471–541 (2011).
[5] Sachdev, S. *Quantum Phase Transitions* (The Press Syndicate of the University of Cambridge, Cambridge, United Kingdom, 1999).
[6] Goldenfeld, N. *Lectures on Phase Transitions and the Renormalization Group* (Addison-Wesley, New York, 1992).
[7] Mott, N. *Metal-Insulator Transitions* (Taylor & Francis, 1990).
[8] Dobrosavljević, V., Trivedi, N. & Valles Jr, J. M. *Conductor Insulator Quantum Phase Transitions* (Oxford University Press, 2012).
[9] A. Georges, G. Kotliar, W. Krauth & M. J. Rozenberg. Dynamical mean-field theory of strongly correlated fermion systems and the limit of infinite dimensions. *Rev. Mod. Phys.* **68**, 13–125 (1996).
[10] Terletska, H., Vučičević, J., Tanasković, D. & Dobrosavljević, V. Quantum critical transport near the Mott transition. *Phys. Rev. Lett.* **107**, 026401 (2011).
[11] Vučičević, J., Terletska, H., Tanasković, D. & Dobrosavljević, V. Finite-temperature crossover and the quantum Widom line near the Mott transition. *Phys. Rev. B* **88**, 75143 (2013).
[12] Furukawa, T., Miyagawa, K., Taniguchi, H., Kato, R. & Kanoda, K. Quantum criticality of Mott transition in organic materials. *Nat. Phys.* **11**, 221–224 (2015).
[13] Pustogow, A. *et al.* Low-temperature dielectric anomaly arising from electronic phase separation at the Mott insulator-metal transition. *npj Quantum Materials* **6**, 9 (2021).
[14] Pustogow, A. *et al.* Rise and fall of Landau's quasiparticles while approaching the Mott transition. *Nature Communications* **12**, 1–8 (2021).
[15] Li, T. *et al.* Continuous Mott transition in semiconductor moiré superlattices. *Nature* **597**, 350–354 (2021).
[16] Pustogow, A. *et al.* Quantum spin liquids unveil the genuine Mott state. *Nature Materials* **17**, 773–777 (2018).
[17] Tan, Y., Dobrosavljević, V. & Rademaker, L. How to recognize the universal aspects of Mott criticality? *Crystals* **12**, 932 (2022).
[18] Dasari, N., Vidhyadhiraja, N. S., Jarrell, M. & McKenzie, R. H. Quantum critical local spin dynamics near the Mott metal-insulator transition in infinite dimensions. *Phys. Rev. B* **95**, 165105 (2017).
[19] Zang, J., Wang, J., Cano, J., Georges, A. & Millis, A. J. Dynamical Mean-Field Theory of Moiré Bilayer Transition Metal Dichalcogenides: Phase Diagram, Resistivity, and Quantum Criticality. *Phys. Rev. X* **12**, 021064 (2022).
[20] Pines, D. & Nozières, P. *The Theory of Quantum Liquids* (Benjamin, New York, 1965).
[21] Brinkman, W. F. & Rice, T. Application of Gutzwiller's variational method to the metal-insulator transition. *Phys. Rev. B* **2**, 4302 (1970).
[22] Deng, X. *et al.* How bad metals turn good: Spectroscopic signatures of resilient quasiparticles. *Phys. Rev. Lett.* **110**, 086401 (2013).
[23] Li, T. *et al.* Continuous mott transition in semiconductor moiré superlattices. *Nature* **597**, 350–354 (2021).
[24] Kotliar, G. *et al.* Electronic structure calculations with dynamical mean-field theory. *Rev. Mod. Phys.* **78**, 865–951 (2006).
[25] Hussey, N. E., Takenaka, K. & Takagi, H. Universality of the Mott-Ioffe-Regel limit in metals. *Philosophical Magazine* **84**, 2847–2864 (2004).
[26] Georges, A., Medici, L. d. & Mravlje, J. Strong correlations from Hund's coupling. *Annual Review of Condensed Matter Physics* **4**, 137–178 (2013).
[27] Pustogow, A. *et al.* Low-temperature dielectric anomaly arising from electronic phase separation at the Mott insulator-metal transition. *npj Quantum Materials* **6**, 1–7 (2021).
